# Internal jugular vein thrombosis by sewing needle ingestion

**DOI:** 10.1016/j.ijscr.2021.105988

**Published:** 2021-05-15

**Authors:** Reda Bendiouri, Ilham Chennoufi, Azeddine Lachkar, Drissia Benfadil, Adil Abdenbi, Fahd Elayoubi, Mohammed Rachid Ghailan

**Affiliations:** a Mohammed First University, Faculty of Medicine and Pharmacy, Oujda, Morocco; b University Hospital Center Mohamed VI, Oujda, Morocco

**Keywords:** Foreign body, Jugular vein thrombosis, Deep neck space infection, Sewing needle, Case report

## Abstract

**Introduction:**

The internal jugular vein thrombosis is usually due to intravenous drug abuse, prolonged central venous catheterization or deep head-neck infections or trauma. Related malignancies, or inflammatory etiologies are described. Our case is interesting by the ingestion of a sewing needle that passes from the pharynx to the internal jugular vein via migration, leading to life-threatening complications: deep neck space infection and internal jugular vein thrombosis.

**Case report:**

We report a case of a 40 years old patient, for acute cervical cellulitis in a context of odynophagia and fever, a CT scan revealed a jugular vein thrombosis, penetrated by a metal density foreign body. The diagnosis of ingested foreign body complicated by cervical cellulitis and thrombosis of the internal jugular vein was made. The patient underwent neck surgery with intravenous antibiotics. The postoperative course was uneventful, after one year of follow-up, no complications have been observed.

**Discussion:**

no consensus has been reached concerning the management of postoperative and post traumatic vein thrombosis. Taking into account the risk of extension of the thrombus and the hemorrhagic risk each case should involve discussions among a multidisciplinary team.

**Conclusion:**

The internal jugular vein thrombosis is a rare complication of ingested foreign bodies which may lead to life threat. The early diagnosis and adequate treatment of its life-threatening complications may result in excellent prognosis.

## Highlights

• No reported cases of internal jugular vein thrombosis caused by ingestion of a sewing needle.
• Association of two life threatening complications: deep neck space infection and internal jugular vein thrombosis
• Our objective was to bring attention to life threatening complications after foreign body ingestion.

## 1 Introduction

Internal jugular vein thrombosis is a rare vascular event that can be defined as the formation of a thrombus located intraluminally in the internal jugular vein. The most common etiologies include prolonged central venous catheterization, intravenous drug use [1,2], ovarian hyperstimulation syndrome [3], infection and malignancy [4,5]. This affection may result in serious complications such as pulmonary embolism and post-thrombotic syndrome. Mortality and morbidity are high in the patients when late and mistaken diagnosis are being made [6]. We related a case report of internal jugular vein thrombosis caused by ingestion of a foreign body. This presentation is interesting by the association of two life threatening complications: deep neck space infection and internal jugular vein thrombosis, due to the ingestion of a sewing needle that passes from the pharynx and extends transversely into the right jugular vein. It has never been previously reported in literature. This case report is in line with the SCARE criteria [7].

## 2 Case report

**Fig. 1:**
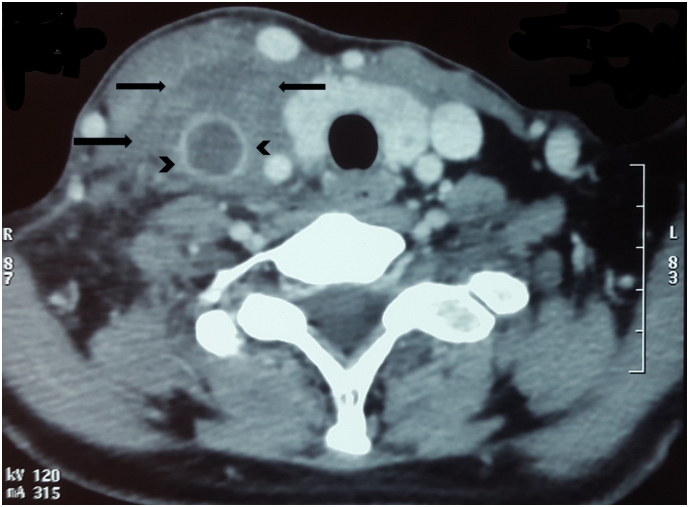
On axial contrast-enhanced computed tomography scan an abnormal low attenuation within the para-pharyngeal space and along the carotid space indicates.

**Fig. 2:**
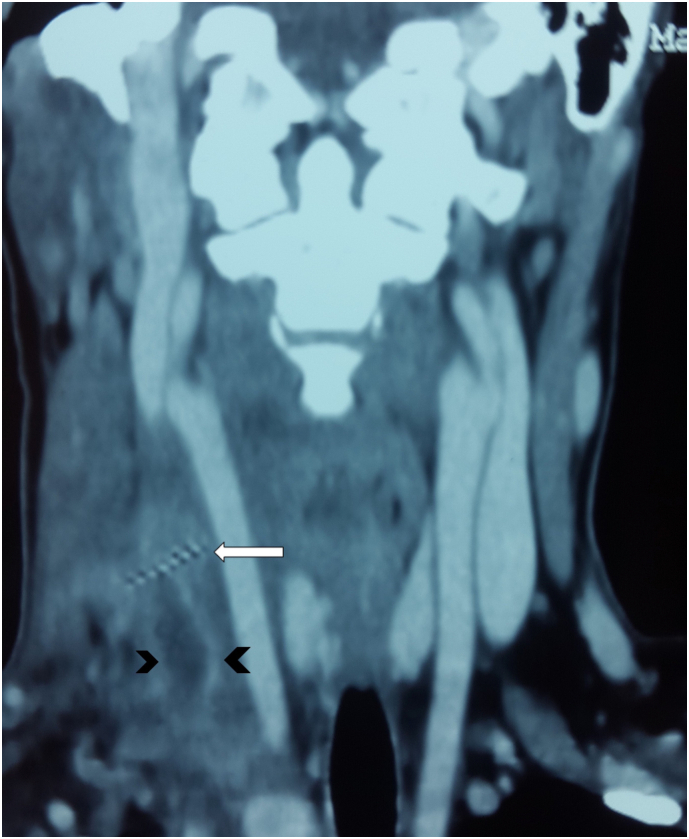
Coronal CECT scan illustrating a jugular vein thrombosis (arrow head) crossed with an oblique metal density foreign body.

**Fig. 3:**
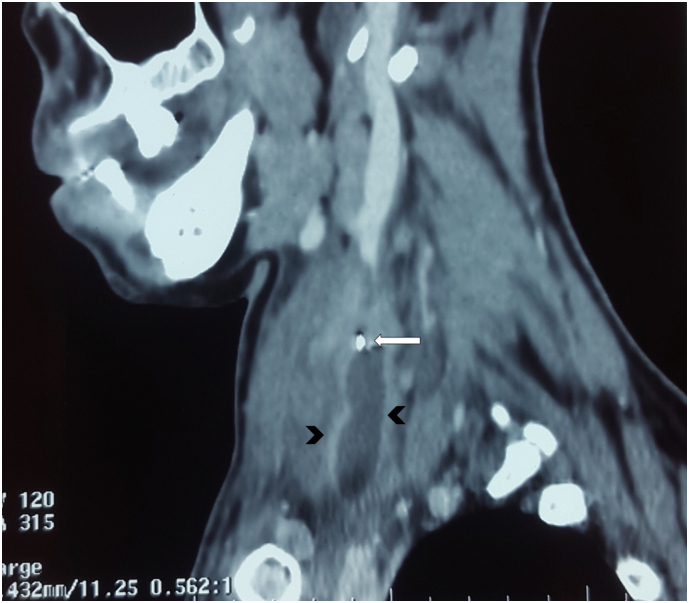
Sagittal CECT scan showing a jugular vein thrombosis (arrow head) penetrated by a metal density foreign body (white arrow).

A 40-year-old woman was admitted for ENT, head and neck surgery emergency care with a 02-week history of fever, pharyngeal foreign body sensation, odynophagia and right-sided neck pain and swelling. She had no clear history of malignancy, central venous catheterization, coagulation disorders, cervical trauma or any foreign body ingestion, genetic and family histories were unremarkable. Physical examination revealed erythematous swelling over the right side of the neck. Nasofibroscopy showed pyriform sinus edema with purulent secretions filling the hypopharynx. Complete blood count (CBC) showed his white blood cell count was (17,000)/mm^3^ with predominant neutrophil, C-reactive protein was 62 mg/l. Contrast-enhanced computed tomography scan revealed Abnormal low attenuation within the parapharyngeal space and along the carotid space indicates cellulitis (Fig. 1), with a vascular filling defect in the left internal jugular vein to left subclavian vein region, penetrated by a metal density foreign body (Fig. 2, Fig. 3). On the basis of the above findings, we diagnosed the patient as having an ingested foreign body complicated by cervical cellulitis and thrombosis of the internal jugular vein.

Because of the cervical cellulitis and the presence of a foreign body, an intravenous probabilistic antibiotic therapy is initiated immediately consisting on third-generation cephalosporins 2 g/24 h, Metronidazole 500 mg/8 h and aminoglycoside 160 mg/24 h, 02 h later a qualified surgeon with 20 years of experience decided to proceed to surgery for better exposure and also effective drainage of fluid collection. Via an incision anterior to the left sternocleidomastoid muscle, the parapharyngeal space was explored and the carotid sheath was opened. A 4 cm sewing needle was found extending transversely into the right jugular vein and the sternocleidomastoid muscle at the level of the thyroid cartilage and thrombosed right internal jugular vein. After removal of the needle, unfractionated heparin was administered intravenously, then the internal jugular vein was ligated proximal and distal to the site of injury. Drainage of abscess cavities and debridement of necrotic tissues were done, and samples were sent for culture. A Penrose drain was inserted, the skin was closed with separate stitches, and systemic heparinization was ceased. The pus culture did not show any organism and the intravenous probabilistic antibiotic therapy was continued. The postoperative course was uneventful, and liquid diet was started on the 3th postoperative day. After a normal barium esophagram, the patient was discharged on the 7th postoperative day and was ordered to complete 02 weeks of oral antibiotic therapy. Within a month the swelling completely disappeared, dysphagia improved, and the patient became normal. The patient is in the 12th month of her follow-up and no complications have been observed during this period.

## 3 Discussion

The main causes of jugular vein thrombosis are prolonged central venous catheterization, intravenous drug use [1,2], ovarian hyperstimulation syndrome [3], infection due to continuous spread and malignancy [4,5]. Traumatic jugular vein thrombosis is an uncommon vascular event [6]. To our knowledge this is the first report of an ingested sewing needle perforating the pharynx, complicated with jugular vein thrombosis and cervical cellulitis. It's unclear how the sewing needle was ingested in this case, the patient did not mention accidental ingestion. She did however perform sewing work at home and often used her lips to hold the needle.

Most studies of deep neck infections recognized pain and swelling of the neck to be the most prevalent symptoms [8]. In our case the foreign body sensation and odynophagia were specific for the unknown ingested foreign body discovered on the CT scan. Ingestion of blunt foreign bodies can cause mucosal ulceration and inflammation. It could lead to intestinal tract perforation, deep neck space infection, mediastinitis, pneumothorax and many other consequences [9].

Radiographic study of the neck is necessary in order to assess the presence, location, size, and the configuration if an ingested object is suspected. It also helps to detect the foreign body induced complications [10,11]. The CT scan is useful for detecting the type of soft-tissue infection, to define the extent of infection, and to provide a reference for surgical procedures [12]. The CT scan also makes a diagnosis of jugular vein thrombosis or other vascular lesions after a foreign body ingestion [13].

The mechanism of venous thrombosis is attributed to Virchow's triad: endothelial damage, hypercoagulability, and alteration of blood flow [14]. In our case we have endothelial damage from an infected sewing needle and altered blood flow resulting from deep neck infection activate clotting factors initiating the thrombotic process.

For patients with jugular vein thrombosis, most authors recommend conservative treatment with intravenous antibiotics and surgical drainage of deep neck infections. Internal jugular vein excision or anticoagulation therapy has not been recommended routinely [14].

Surgical thrombectomy in central veins is technically difficult and would be reserved for cases refractory to intravenous antibiotics and anticoagulation treatment [15]. In our case we ligated the right internal jugular vein because of its laceration with a sewing needle, to avoid the extension of the thrombus and the bleeding risks.

When the jugular vein thrombosis is associated with deep neck infection It is accepted that infection must be treated using antibiotics against anaerobic, Streptococcus and Staphylococcus bacteria. Beta- lactamic antibiotics associated with beta-lactamase inhibitors are recommended as the first treatment option [16,17].

## 4 Conclusion

The internal jugular vein thrombosis is a rare complication of ingested foreign bodies which may lead to life threat. Based on our case report, we recommend the CT scan imaging in all patients with acute cervical cellulitis to define the extent of the infection, the possible causes of infection, with consideration of advanced complications such as the migration of the sewing needle from the pharynx to the internal jugular vein, which lead to thrombosis in this vein. The anticoagulant therapy and the length of antibiotic treatment in post-traumatic jugular vein thrombosis remain controversial. Surgical treatment is recommended in case of deep neck space infections, abscess formation, septic embolism or large septic thrombosis.

## Funding

We have any financial sources for our research.

## Ethical approval

The study committee of the university hospital center approves the favorable opinion to publish this work.

## Consent

Written informed consent was obtained from the patient for publication of this case report and accompanying images. A copy of the written consent is available for review by the Editor-in-Chief of this journal on request.

## Author's contribution

Dr.RB, Dr.IC, Dr.AL, Dr.DB, Dr.AA, Dr.FE have analysed and performed the literature research, Pr. RG performed the examination and performed the scientific validation of the manuscript. Dr.Reda BENDIOURI was the major contributor to the writing of the manuscript. All authors read and approved the manuscript.

## Registration of research studies

Not applicable.

## Guarantor

Dr. BENDIOURI REDA.

## Provenance and peer review

Not commissioned, externally peer-reviewed.

## Availability of data and material

The datasets in this article are available in the repository of the ENT database, Chu Mohamed VI Oujda, upon request, from the corresponding author.

## Declaration of competing interest

All authors disclose any conflicts of interest.
